# Neonatal Duodenal Obstruction: A 15-Year Experience

**Published:** 2016-04-10

**Authors:** Kamal Nain Rattan, Jasbir Singh, Poonam Dalal

**Affiliations:** 1 Department of Pediatric Surgery, PGIMS, Rohtak, Haryana India 124001; 2 Department of Pediatrics, PGIMS, Rohtak, Haryana India 124001

**Keywords:** Duodenal atresia, Annular pancreas, Duodenal obstruction, Malrotation, Neonate

## Abstract

Background: Congenital duodenal obstruction is one of the commonest causes of neonatal intestinal obstruction. We are presenting our 15-year experience by analyzing clinical spectrum and outcome in neonates with duodenal obstruction admitted at our center.

Material and Methods: The hospital records of all neonates admitted with duodenal obstruction from June 2000 to June 2015 were reviewed. The patient records were analyzed for antenatal diagnosis, age, sex, clinical presentation, diagnosis, associated anomalies, surgical procedures performed; postoperative morbidity and mortality. We excluded from our study malrotation of gut associated with congenital diaphragmatic hernia and abdominal wall defects.

Results: A total of 81 patients were admitted, out of which 56 were males and 25 were females. Polyhydramnios was detected in 24 (30%) pregnancies. Average birth weight was 2.1±1.0Kg and average gestational age was 38 (SD±1) weeks with 17 (21%) preterm neonates. Presenting features were vomiting in 81(100%) which was bilious in 81% and non-bilious in 19%, epigastric fullness in 56 (69%) and dehydration in 18 (22%) and failure to thrive in 16 (19%). Most common cause of obstruction was duodenal atresia in 38 (46.9%), followed by malrotation of gut in 33 (40.7%), and annular pancreas in 4 cases. Depending upon site of location, infra-ampullary obstruction was the most common in 64 (79%), supra-ampullary in 9 (7.4%) and ampullary 8 neonates. Both duodenal atresia and malrotation of gut was present in 4 cases. X-ray abdomen was most commonly used investigation to confirm the diagnosis. All cases were managed surgically by open laparotomy. Eleven (13.5%) patients died due to sepsis and associated congenital anomalies.

Conclusion: Congenital duodenal obstruction most commonly presents in early neonatal period with features of upper GIT obstruction like vomiting and epigastrium fullness as in our series. Early antenatal diagnosis and surgical interventions hold the key in achieving good outcome. Associated congenital anomalies, prematurity, sepsis and delayed presentation are the main risk factors for post-operative mortality and morbidity.

## INTRODUCTION

Congenital duodenal obstruction is the one of the commonest cause of neonatal intestinal obstruction accounting for almost 50% cases [1]. The reported incidence of duodenal obstruction is 1 in 2500 to 10000 live births [2, 3]. Duodenal obstruction may be classified as intrinsic or extrinsic and both and it may be complete or partial [4-7]. Common causes are duodenal atresia, malrotation, and rarely annular pancreas. We herein describe our 15-year experience of managing duodenal obstruction, owing to various causes, in neonates.

## MATERIALS AND METHODS

It was a retrospective study conducted at our tertiary care hospital which deals mostly rural population. We had analyzed available hospital records of neonates admitted with duodenal obstruction at our center from June 2000 to June 2015. We analyzed the collected data according antenatal diagnosis, demographic criteria, age, gender, time of presentation, clinical presentations, associated anomalies, type of surgery done, and outcome. All of the patients were followed up for one year duration. We excluded malrotation of gut associated with congenital diaphragmatic hernia and abdominal wall defects.

All of the neonates were referred to us from peripheral health centers for various problems. After hemodynamically stabilizing the patients, they were investigated to find out underlying cause. The diagnosis was confirmed with plain X-ray abdomen (erect) which showed double bubble appearances in duodenal atresia (Fig. 1) X ray abdomen (erect) was the most important investigation leading to confirmation of diagnosis of duodenal obstruction. Ultrasonography (USG) abdomen was done to rule out associated congenital anomalies. It also confirmed and showed whirlpool sign in malrotation of gut. When clinical examination was suggestive, ECHO was also done to rule out associated congenital heart diseases (CHD). Radiological contrast studies were done to confirm the diagnosis of malrotation and type I duodenal atresia with partial obstruction. In neonates with suspected Down syndrome, karyotyping was used to confirm the trisomy and thyroid profile and hearing assessment were also done during follow up which came out normal. Neonates with ARM, underwent colostomy. In internal herniation derotation of gut was done followed by correction of underlying cause.

**Figure:**
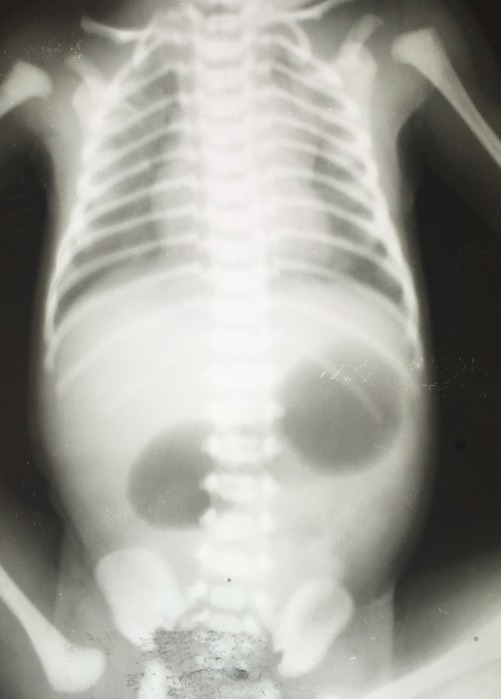
Figure 1: double bubble sign

## RESULTS

Demography: A total of 81 patients were admitted during study period with 56 neonates were male and 25 were female with M:F= 2.2:1. Mean gestational age observed in study was 38 SD±1 weeks with 64 neonates as fullterm and mean birth weight was 2.1±1.0 Kg. Antenatal supervision was poor in our study and there was history of polyhydramnios in 24 (30%) pregnancies.

Presentation: Most common presenting feature was vomiting in all of 81 (100%) case (non-bilious in 19% and bilious in 81%), epigastric fullness was seen in 56 (70%), dehydration in 18(22%) neonates. Fifteen (18.5%) neonates were presented with shock. we found that 22 (27%) presented with in first 48 hours of life. Thirty (37%) neonates presented more than 10 days of life. Physiological hyperbilirubinemia was seen in 14 neonates for which phototherapy was given.

Surgery: After stabilizing the fluid and electrolyte imbalance, all of them were taken for surgery. Duodenoplasty with web resection was done in duodenal web, duodenoduodenostomy in type I and II atresia and duodeno-jejunostomy in type III atresia. Ladd's procedure was done in malrotation. In 2 patients malrotation of gut was associated with gangrenous changes, so derotation of gut was done and abdomen was closed. 

Cause of obstruction was duodenal atresia in 38 (46.9%), malroation of gut in 33 (40.7%), both malrotation and atresia was present in 6 cases and annular pancreas in 4. Type I atresia was the commonest to occur in 18, type II atresia in 12 and type III atresia was seen in 8 neonates. Multiple jejunal and ileal atresias were present in 4 patients who were diagnosed with malrotation. They were managed by open laparotomy and resection of the atretic segment of gut with jejunoileal anastomosis. Outcome in all these 4 neonates was good as after average seven days stay in NICU, they were started oral feed and discharged after 14 days stay.

Associated anomalies: While analyzing associated anomalies, congenital cardiac diseases (CHD) was present in 7 neonates, Down`s syndrome was seen in 6 cases of duodenal atresia and it was confirmed by karyotyping. Anorectal malformation (ARM), lumber hernia and solitary kidney were present in 1 patient each (Table 1).

**Figure:**
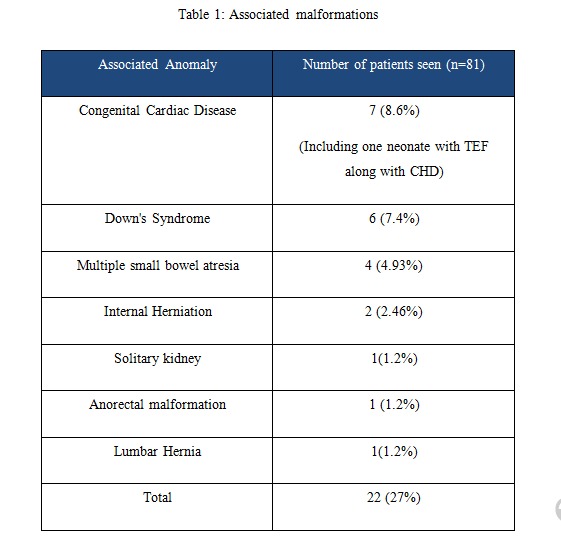
Table 1: Associated malformations

Postoperative course: After surgery all babies were kept in NICUs under monitoring. All cases were started orally when the baby passed stool and there was no bilious aspirates. Average period to start oral feeds was 5 days. Once babies started tolerating full feeds and gaining weight, they were discharged. Average postoperative hospital stay was of 14±4 days. After discharge, all neonates were followed for one year. On follow up six patients were admitted again, 4 of them managed conservatively and 2 required repeat surgery for adhesive lesions. 

Mortality: During postoperative period 11 (13.5%) patients were expired. Sepsis was responsible in 50 % cases, associated congenital anomalies in 4 neonates. Two cases of malrotation who presented with septic shock with midgut gangrene expired due to septic shock. Among eleven neonates who expired, 63.6 % were preterm.

## DISCUSSION

Duodenal obstruction can present at variable age depending whether obstruction is partial or complete. Complete obstructive lesions like duodenal atresia present earlier as compared to partial obstruction like malrotation, annular pancreas and duodenal diaphragm [3]. Mean age of presentation in our study was 5±2 day of life. Similarly, Zamir et al reported mean age of presentation as 4 days [7]. While describing clinical presentations, vomiting was the most common presentation in all 81 (100%) neonates. Non bilious vomiting will be seen infra-ampullary obstruction which was seen in 9 neonates. So we should keep duodenal obstruction as differential diagnosis even in presence of non-bilious vomiting.

Duodenal atresias (DA) were observed in 38(46.9%) patients and were the most common cause of obstruction. There are increased chance of preterm babies to get affected more with duodenal atresia as out of 38 neonates 10 (26.3%) were preterm. Escobar had similar findings in their study with 169 neonates, where 37% were preterm [5]. As reported in literature, Type I atresia was the most common to be seen in 18 neonates. Malrotation of gut was also leading cause of obstruction in our study seen in 33 (40%) patients. Malrotation with midgut volvulus can results in gangrenous changes due to vascular compromise leading to increased mortality and short bowel syndrome following resection of gangrenous gut. But in our series two cases who presented with complete midgut volvulus died due to septic shock.

Duodenal atresia had been reported to be associated with other significant congenital anomalies with incidence of 38-55% [6]. Spectrum of various congenital anomalies includes Down's syndrome, cardiac defects, esophageal atresia, urinary tract malformation and other gastrointestinal anomalies. We had observed associated anomalies in 22 (27%) neonates. Cardiac defects were the most common anomalies reported in 7 (8.6%). Down's syndrome was next most common anomaly seen in 6 (7.4%). All of 6 patients were diagnosed with duodenal atresia type II. Chen et al had reported incidence of congenital anomalies 50.5% with cardiac defects as most common [8]. Internal herniation of gut was seen in 2 patients in malrotation. Solitary kidney, anorectal malformation (ARM) and lumber hernia were present in 1 neonate each. It had been reported that presence of Down's syndrome also increases the risk of CHDs.

Overall mortality observed in our series was of 11 (9%) patients. Sepsis was the most important risk factor leading to 50% neonatal mortality. Four (40%) of our neonates who died were having associated anomalies like congenital heart diseases. Other presumable reasons were preterm gestation, low birth weight and delayed diagnosis. In a review of 287 neonates, Chen et al observed overall mortality of 5.9% [8]. In another study by Kaddah et al reported mortality was 21.1% [9]. Zamir et al had reported mortality of 58% in a series from Pakistan [7]. Postoperative survival rate in duodenal obstruction is gradually improving over last years. In a similar study done at our institute two decades earlier, mortality was reported 39% [10]. In western counties where antenatal supervision is good and neonates are diagnosed early, postoperative outcome of duodenal obstruction is much better than developing countries [11,12].

## CONCLUSION

Congenital duodenal obstruction is one of the commonest anomalies of neonates. It can present with a varied clinical picture like frequent vomiting, epigastrium fullness and failure to gain weight in neonate. Early diagnosis and interventions holds the key to have the good outcome congenital duodenal obstruction. Delayed diagnosis, associated congenital anomalies, prematurity with low birth weight and sepsis are important risk factors associated with increased mortality and morbidity. Advances in pediatric surgical techniques and NICUs have resulted in nearly 100% postoperative survival.

## Footnotes

**Source of Support:** None

**Conflict of Interest:** None
